# Detection of gray matter microstructural changes in Alzheimer’s disease continuum using fiber orientation

**DOI:** 10.1186/s12883-020-01939-2

**Published:** 2020-10-02

**Authors:** Peter Lee, Hang-Rai Kim, Yong Jeong, for the Alzheimer’s Disease Neuroimaging Initiative

**Affiliations:** 1 Department of Bio and Brain Engineering, Korea Advanced Institute of Science and Technology, Daehak-ro 291, Yuseong-gu, Daejeon, 34141 Republic of Korea; 2 KI for Health Science and Technology, Korea Advanced Institute of Science and Technology, Daejeon, Republic of Korea; 3 Graduate School of Medical Science and Engineering, Korea Advanced Institute of Science and Technology, Daejeon, Republic of Korea

**Keywords:** Alzheimer’s disease, Early diagnosis, Diffusion tensor imaging, Microstructure, Biomarkers

## Abstract

**Background:**

This study aimed to investigate feasible gray matter microstructural biomarkers with high sensitivity for early Alzheimer’s disease (AD) detection. We propose a diffusion tensor imaging (DTI) measure, “radiality”, as an early AD biomarker. It is the dot product of the normal vector of the cortical surface and primary diffusion direction, which reflects the fiber orientation within the cortical column.

**Methods:**

We analyzed neuroimages from the Alzheimer’s Disease Neuroimaging Initiative (ADNI) database, including images from 78 cognitively normal (CN), 50 early mild cognitive impairment (EMCI), 34 late mild cognitive impairment (LMCI), and 39 AD patients. We then evaluated the cortical thickness (CTh), mean diffusivity (MD), which are conventional AD magnetic resonance imaging (MRI) biomarkers, and the amount of accumulated amyloid and tau using positron emission tomography (PET). Radiality was projected on the gray matter surface to compare and validate the changes with different stages alongside other neuroimage biomarkers.

**Results:**

The results revealed decreased radiality primarily in the entorhinal, insula, frontal, and temporal cortex with further progression of disease. In particular, radiality could delineate the difference between the CN and EMCI groups, while the other biomarkers could not. We examined the relationship between radiality and other biomarkers to validate its pathological evidence in AD. Overall, radiality showed a high association with conventional biomarkers. Additional ROI analysis revealed the dynamics of AD-related changes as stages onward.

**Conclusion:**

Radiality in cortical gray matter showed AD-specific changes and relevance with other conventional AD biomarkers with high sensitivity. Moreover, radiality could identify the group differences seen in EMCI, representative of changes in early AD, which supports its superiority in early diagnosis compared to that possible with conventional biomarkers. We provide evidence of structural changes with cognitive impairment and suggest radiality as a sensitive biomarker for identifying early AD.

## Background

Alzheimer’s disease (AD) is notorious for its long preclinical period where various pathophysiological changes occur before the main symptoms develop. As the progression of AD is not completely understood, early diagnosis and intervention remain challenging [1, 2]. Repetitive failures of recent drug trials are attributed to the application of treatment to patients with relatively advanced disease [3–5]. Thus, identification of people in the earlier stages of pathology is critical in clinical trials and may be promising for controlling this devastating disease.

At present, several biomarkers are used to diagnose and monitor disease progression: amyloid and tau deposits based on positron emission tomography (PET) imaging or cerebrospinal fluid (CSF) samples, volumetric and morphologic analysis using T1-weighted magnetic resonance imaging (MRI) and clinical assessments. Although the results from PET and CSF screening are promising, these interventions are more invasive than MRI. In the search for suitable MRI biomarkers, researchers have focused on characterizing early mild cognitive impairment (EMCI) and late mild cognitive impairment (LMCI) [6, 7]. Although the criteria for separating EMCI and LMCI are based on scores on memory tests, biomarkers in individuals with EMCI show a continuous spectrum to those in individuals with LMCI, implying that EMCI is a transitional stage of AD [8]. Thus, evaluating sequential changes in EMCI and LMCI should help in understanding early AD.

Diffusion tensor imaging (DTI) utilizes the diffusion of water molecules within tissues and provides axonal microstructural properties; thus, it is widely applied when studying white matter integrity [9, 10]. Early AD studies using DTI have mainly focused on the white matter. However, since white matter changes in AD may be the result of Wallerian degeneration, followed by neurodegeneration in the gray matter [11, 12], the destruction of white matter is a less sensitive change in AD.

The idea of measuring microstructural changes in gray matter using DTI has been demonstrated in both AD and frontotemporal dementia [13–15]. These studies showed that gray matter mean diffusivity (MD) is higher in patients than in healthy controls and that MD could be a promising imaging biomarker. However, there is a lasting notion that increased MD could be overestimated by the CSF signal, and this effect persists even with rigorous correction approaches, such as partial volume effects correction [16].

To overcome this problem, we adopted radiality, which presumably reflects the integrity of tangential cortical fibers. In an initial study of the applicability of radiality in AD, we sought to find an association with conventional MRI biomarkers [17]. A recent study investigated the association between anisotropic diffusion and cortical structures through postmortem diffusion MRI along with histology in multiple sclerosis [18]. Although this study was limited to observing certain brain regions, it provides relevant evidence to measure cortical changes with DTI. Moreover, this parameter has been applied to study neurodevelopment and can distinguish the stages of aging [19–21]. Cortical microstructural changes are often observed with aging or neurodegeneration, which can be viewed as the opposite of neurodevelopment [22–25]. Thus, changes in fiber orientation may suggest cortical alterations and could be used as a biomarker in neurodegenerative diseases.

In this study, we hypothesized that the radiality within gray matter could be a microstructural measure of the cortex and used as the early signature of AD. We performed a cross-sectional surface-based cortical analysis approach using DTI, amyloid PET, and tau PET images to the AD continuum [17]. We evaluated whether gray matter radiality shows: i) early mesoscopic AD-related pathological change, and ii) complementarity with conventional AD biomarkers while providing distinct information regarding AD-related pathologies.

## Methods

### Demographics

Data used in this study were obtained from the Alzheimer’s Disease Neuroimaging Initiative (ADNI) (adni.loni.usc.edu). The ADNI was launched in 2003 as a public-private partnership, led by Principal Investigator Michael W. Weiner, MD. The primary goal of ADNI has been to test whether serial magnetic resonance imaging (MRI), positron emission tomography (PET), other biological markers, and clinical and neuropsychological assessment can be combined to measure the progression of mild cognitive impairment (MCI) and early Alzheimer’s disease (AD). For up-to-date information, see www.adni-info.org.

From the ADNI database, we analyzed subjects who underwent both MRI and PET (amyloid, AV 45 and tau, AV 1451) including 78 cognitively normal (CN), 50 EMCI, 34 LMCI, and 39 AD individuals. Subjects were sampled according to the following criteria: age, around 60 to 90 years old, education, 12 to 20 years, and gender-matched within groups. To assess the AD continuum, amyloid-negative CN and amyloid-positive EMCI, LMCI, and AD subjects were selected. The EMCI group was subdivided into 38 dementia non-converters (stable EMCI) and 12 converters to assess changes in disease progression. A total of 201 subjects’ T1 and DTI images were gathered from the ADNI. To increase the sample size, a multi-center approach was used, as discussed in [13]. The amyloid positivity of subjects was determined using whole brain PET AV45 standardized uptake value ratio (SUVR) with a 1.11 cutoff. Table 1 shows the demographics of the subjects used in this study; note that 44 CN subjects, nine EMCI subjects, five LMCI subjects, and three AD subjects underwent AV1451 tau PET imaging. An additional 28 CN subjects with amyloid positivity were analyzed to identify the earliest AD pathological changes as presented in Supp**.** Table 1.

**Table 1:** Demographics

	CN (***n*** = 78)	EMCI (***n*** = 50)	EMCI Non-converter (***n*** = 38)	EMCI Converter (***n*** = 12)	LMCI (***n*** = 34)	AD (***n*** = 39)	Post hoc
**Female, n (%)**	42 (53.8)	19 (37.2)	14 (36.8)	5 (41.7)	15 (44.1)	17 (43.6)	–
**Age (SD) (y)**	72.7 ± 5.9	74.7 ± 5.3	74.1 ± 4.9	76.4 ± 4.7	73.9 ± 5.6	74.7 ± 7.2	–
**Education (SD) (y)**	16.7 ± 2.5	15.2 ± 2.6	15.0 ± 2.5	15.6 ± 3.1	16.1 ± 2.8	15.4 ± 2.9	–
**GCDR (SD)**	0.0	0.5	0.5	0.5	0.5	0.8 ± 0.3	CN < EMCI = LMCI<AD
**MMSE (SD)**	29.3 ± 1.5	28.2 ± 1.2	28.3 ± 1.1	28.1 ± 1.7	27.6 ± 1.4	24.4 ± 4.0	CN > EMCI = LMCI>AD
**MADAS-Cog (SD)**	9.7 ± 6.8	13.6 ± 5.9	13.2 ± 5.0	14.5 ± 4.9	14.6 ± 4.8	26.3 ± 14.2	CN < EMCI = LMCI<AD
**Immediate recall (SD)**	14.2 ± 2.9	10.4 ± 3.4	10.5 ± 3.6	9.9 ± 2.7	6.4 ± 3.3	3.8 ± 2.0	CN > EMCI>LMCI>AD
**Delayed** **recall (SD)**	12.8 ± 3.4	8.6 ± 2.0	8.6 ± 2.1	8.7 ± 1.6	3.1 ± 2.7	1.3 ± 1.6	CN > EMCI>LMCI>AD
**MRI center**	30/48	40/10	29/9	11/1	28/6	36/3	–
**Florbetapir+,** **n (%)**	0 (0)	50 (100)	38 (100)	12 (100)	34 (100)	39 (100)	–
**AV1451 image,** **n (%)**	44 (68.8)	9 (14.1)	8 (21.1)	1 (8.33)	5 (7.8)	3 (4.7)	–

Data are n (%) or mean ± SD values. There were no gender, age, or year of education intergroup differences. GCDR, MMSE, and MADAS-Cog scores in EMCI and LMCI did not show significant differences. Analysis of variance with Tukey test was used for post hoc analysis with *p* < 0.05. For MRI data, two major scanners were used: GE and SIEMENS and delineated as MRI center GE/SIEMENS

*AD* Alzheimer’s disease, *CN* Cognitively normal, *EMCI* Early mild cognitive impairment, *GCDR* Global Clinical Dementia Rating, *LMCI* Late mild cognitive impairment, *MADAS-Cog* Modified Alzheimer’s Disease Assessment Scale-Cognitive subscale, *MMSE* Mini Mental State Examination

### Image processing

**Fig. 1:**
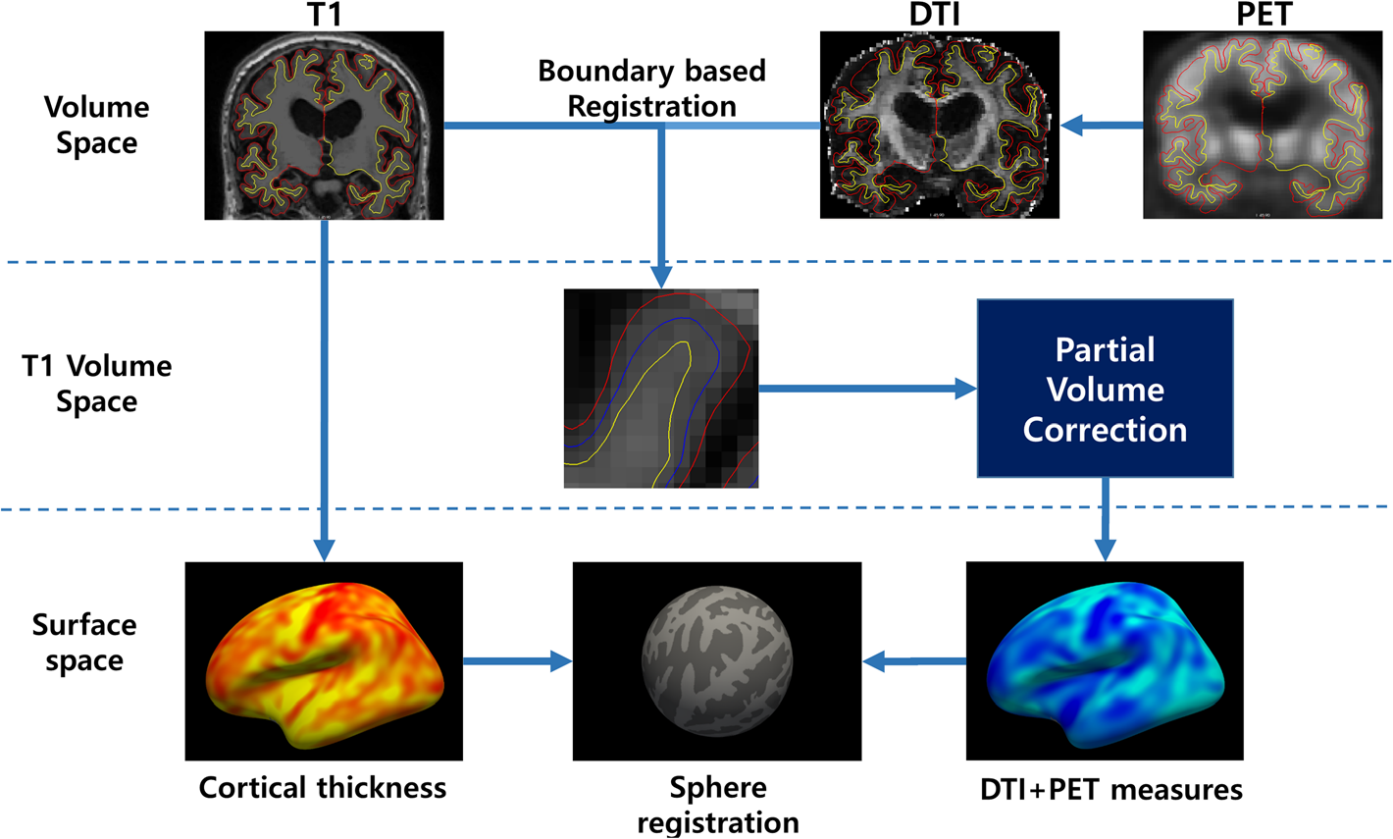
Overall scheme for surface projection analysis. DTI and PET images were boundary-based registered to the T1 image and projected to the fsaverage surface for group comparison

T1-weighted images were processed using FreeSurfer package v6.0 (http://surfer.nmr.mgh.harvard.edu) as previously reported in [13]. Cortical thickness (CTh) maps were registered to the FreeSurfer average sphere using spherical registration for group comparison. DTI and PET images were registered with respect to T1 images using a boundary-based algorithm for further processing. DTI images were processed using the FSL package as follows: eddy current correction, rotate gradient vectors from the results of eddy correction, and tensor fitting to produce the MD map and primary eigenvector map. DTI metrics were further processed to avoid partial volume effects following Koo et al. [26]. PET images were partial volume corrected using mri_gtmpvc which is built into the FreeSurfer package. PET images were normalized by mean signal from the whole cerebellum and converted to SUVR for amyloid and tau PET, AV45, and AV1451, respectively. The images were then boundary-based registered to corresponding T1 structural images. To avoid any partial volume effects, the center parts of the cortical column were sampled for surface analysis. Lastly, CTh was smoothed with a 10-mm full width half maximum Gaussian kernel, while other modalities were smoothed with a 15-mm kernel. Figure 1 shows an overall schematic of the process.

### Calculation of radiality

A surface normal vector was obtained from the individual gray matter surface to define the cortical orientation. FreeSurfer represents the surface in triangular meshes, and the surface normal vector can be computed using the cross-product between edges. The vertex-wise dot product between the primary diffusion direction, primary eigenvector of the diffusion tensor, and the surface normal vector was quantified as a radiality index, *r*, where *v* represents the surface normal vector and *e*_1_ represents the primary diffusion direction [22].

$$ r=\left|{\hat{v}}_n\cdot {\hat{e}}_1\right| $$

It ranges from 0 to 1, where *r* = 0 indicates tangential diffusion and *r* = 1 indicates radial diffusion to the cortex. The subject’s principal eigenvector map was projected onto the individual surface reconstruction to calculate vertex-wise radiality, as discussed in [22].

### Cutoff analysis

To further test the feasibility of radiality as an AD biomarker, we performed cutoff analysis using receiver operating characteristic graphs to distinguish CN from different AD stages, as shown in Supp. Table 2**.** The feature used was the mean radiality within the cluster obtained from the CN vs. EMCI group comparison. By varying the cutoff, we sought to find the cost-effective point where it minimizes the difference between sensitivity and specificity [27].

### Statistical analysis

We first compared the differences between groups for radiality, CTh, MD, AV45, and AV1451 using a general linear model, which is available in FreeSurfer. The results were cluster-wise corrected for a family-wise error (FWE)-corrected *p*-value < 0.05.

To test the associations between radiality and other neuroimaging biomarkers, we calculated a set of vertex-wise partial correlations with radiality as the dependent variable and CTh, MD, AV45, and AV1451 as the independent variable. Age, gender, years of education, and MRI center were set as covariates for cluster analyses. A permutation test was applied to account for multiple comparisons using a Monte Carlo simulation with 10,000 repeats, which is a built-in function of FreeSurfer.

To test the linear relationship between radiality and other neuroimaging biomarkers, we quantified mean metrics within AD-specific ROIs. ROIs include the entorhinal, fusiform, insula, inferior, middle, and superior temporal cortex. Mean metrics within ROIs were plotted in box and whisker plots and are presented in Fig. 5e and Fig. 6. Significant differences between groups were tested using one-way analysis of variance (ANOVA).

## Results

### Group comparison along AD continuum

**Fig. 2:**
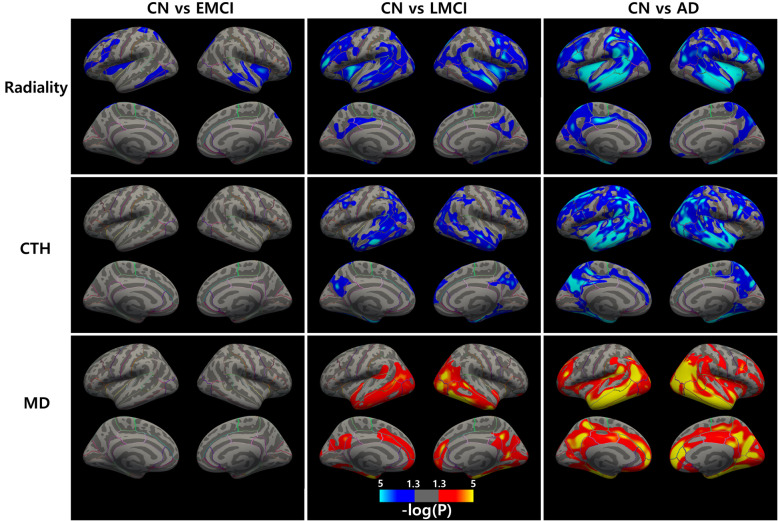
Group differences in radiality, cortical thickness, and mean diffusivity. From left to right: CN vs. EMCI, CN vs. LMCI, and CN vs. AD. The blue cluster shows a decrease in metrics and the red cluster shows an increase in metrics. All clusters were FWE corrected for *p* < 0.05. Color bar indicates *p*-value interval of 0.05 to 10^− 5^

We first compared radiality, CTh, and MD differences between groups, i.e., CN vs. EMCI, CN vs. LMCI, and CN vs. AD. The results were cluster-wise corrected for a FWE-corrected *p* < 0.05. Figure 2 shows the significant group different clusters ranging from a *p*-value of 0.05 to 10^− 5^. Only radiality could delineate the difference between EMCI and CN. Compared to CN, all groups showed decreased radiality, decreased CTh, and increased MD. There was no group difference in radiality between EMCI and LMCI.

### EMCI non-converter versus converter

**Fig. 3:**
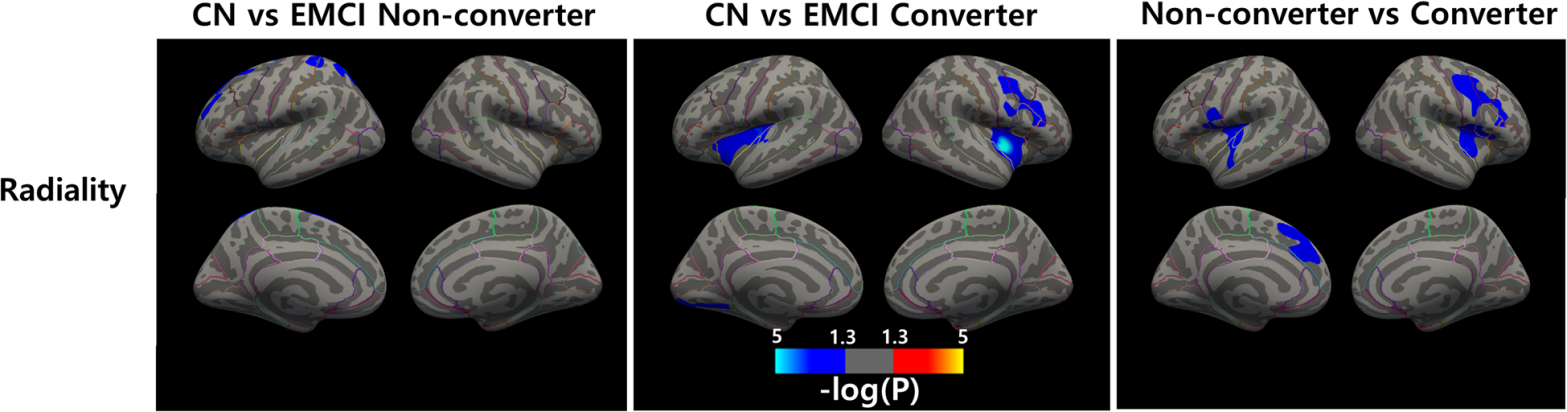
Differences in radiality between EMCI non-converter and converter. From left to right: CN vs. EMCI non-converter, CN vs. EMCI converter, and non-converter vs. converter. The blue cluster shows a decrease in metrics and the red cluster shows an increase in metrics. All clusters were FWE corrected for *p* < 0.05. Color bar indicates *p*-value interval of 0.05 to 10^− 5^

We compared radiality between CN and EMCI non-converter, CN and EMCI converter, and EMCI non-converter vs. converter. The results were cluster-wise corrected for a FWE-corrected *p* < 0.05. Figure 3 shows significant group different clusters ranging from a *p*-value of 0.05 to 10^− 5^. Compared to CN, the EMCI non-converter showed decreased radiality in the left superior frontal and superior parietal cortices. The EMCI converter showed decreased radiality mainly in the bilateral insula cortex. Direct comparison between EMCI non-converter and converter delineated the bilateral insular, left superior frontal, and right precentral cortex.

### Partial correlation between radiality and other imaging biomarkers

**Fig. 4:**
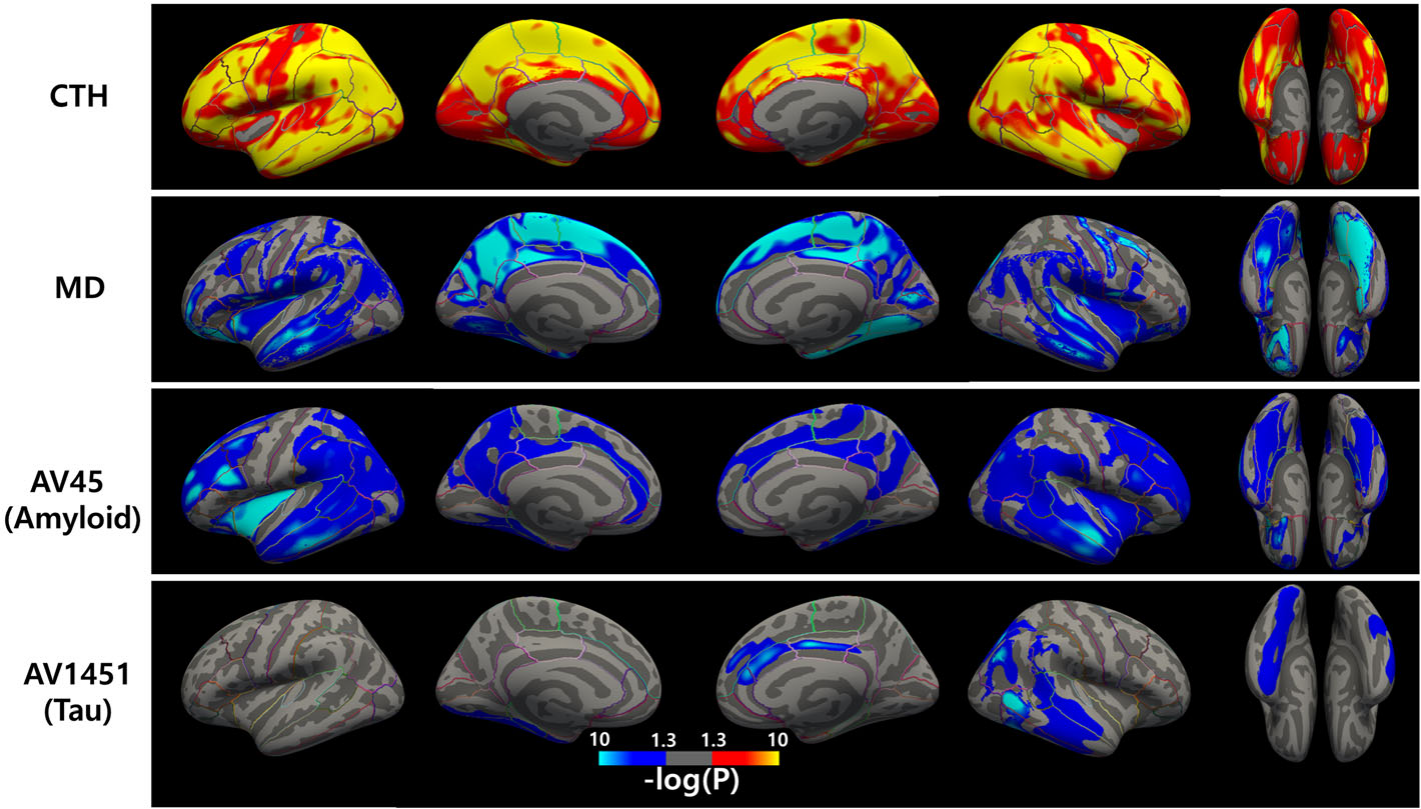
Partial correlation between radiality and image biomarkers. The red cluster shows a positive correlation with radiality, and the blue cluster shows a negative correlation. Cortical thickness showed positive correlations, and mean diffusivity, AV45, and AV1451 showed negative correlations. Color bar indicates *p*-value interval of 0.05 to 10^− 10^

We then found a vertex-wise correlation between radiality and other imaging biomarkers, as shown in Fig. 4. CTh showed mostly positive correlations with decrease in cortical thickness accompanied by a decrease in radiality. MD mostly negatively correlated with an increase in MD accompanied by a decrease in radiality. Amyloid and tau levels negatively correlated with radiality.

### Correlations between radiality and other imaging biomarkers

**Fig. 5:**
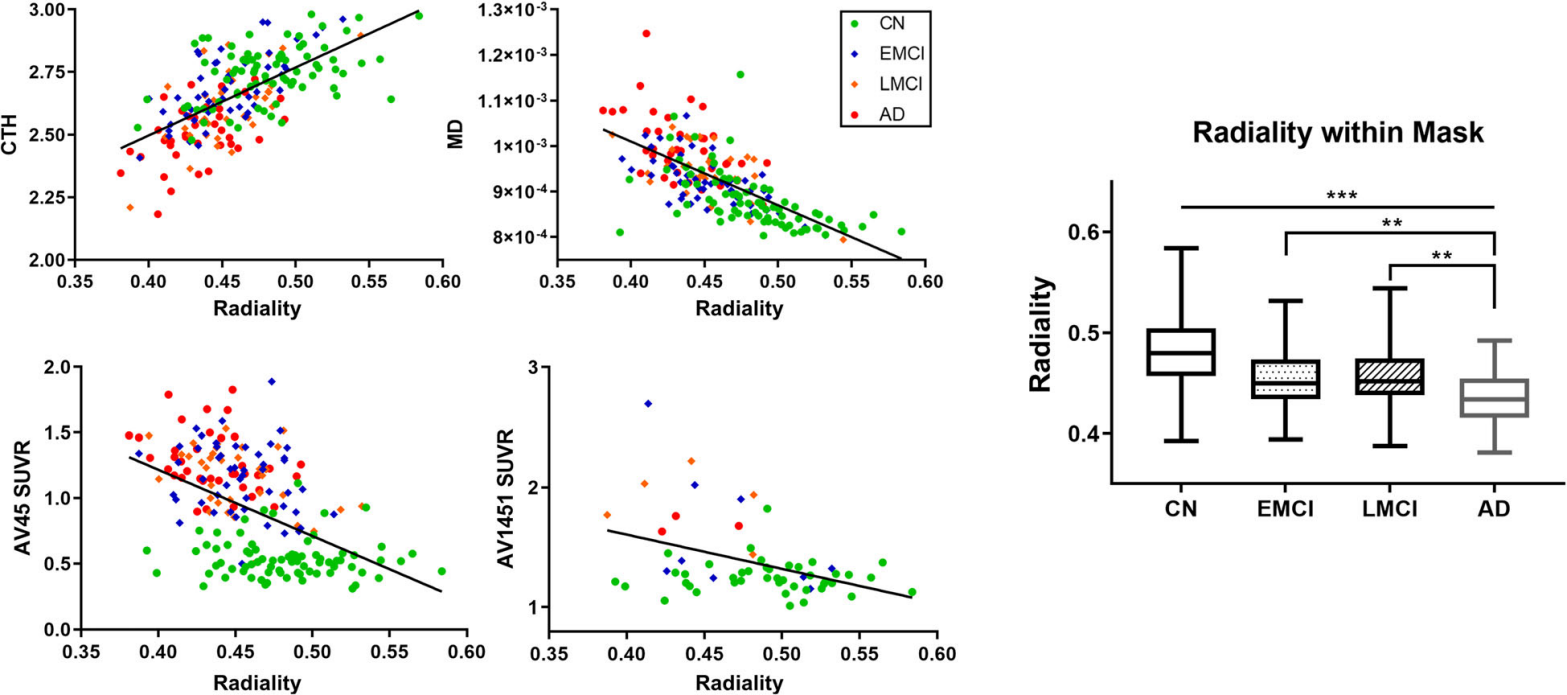
Correlation between radiality with other biomarkers. **a**-**d** Scatter plot between image biomarkers and radiality. Radiality showed high association with conventional biomarkers, indicating that it reflects neuropathology of AD. **a** CTh, **b** MD, **c** AV45, **d** AV1451. **e** Box plot of group radiality comparison within AD-specific ROIs. Radiality from CN showed significant differences with that in EMCI, LMCI, and AD, *p* < 0.001. There were no differences between EMCI and LMCI

In order to find progressive changes in radiality reflective of disease progression, an AD-specific ROI mask was used to calculate mean biomarker data. Each subject’s mean data were scatter-plotted and used to calculate the Pearson correlation, as shown in Fig. 5. The correlation for CTh was *R* = 0.641, that for MD was *R* = − 0.677, that for AV45 was *R* = − 0.490, and that for AV1451 was *R* = − 0.412 with radiality.

### Radiality dynamics from AD-specific ROIs

**Fig. 6:**
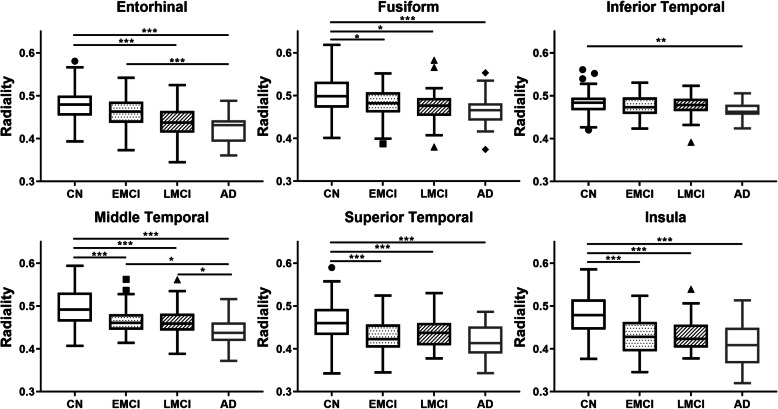
Box plot of group radiality comparison within AD-specific ROI. **a** Entorhinal, **b** fusiform, **c** inferior temporal, **d** middle temporal, **e** superior temporal, **f** insular cortex. Significance was tested using one-way ANOVA; * *p* < 0.05, ** *p* < 0.01, *** *p* < 0.001

To find generative changes in radiality during disease progression, the mean radiality in AD ROIs was calculated for direct comparison between groups. Radiality within AD-specific ROIs was plotted in a box and whisker plot, as shown in Fig. 6. The results showed decreasing radiality with disease progression. Significance was tested with one-way ANOVA with *p*-values < 0.05, 0.01, and 0.001. The insula, middle, and superior temporal cortex showed the most radiality reduction with disease progression.

### Cutoff analysis using radiality

Classification of CN vs. EMCI showed 70.5% accuracy with 70.2% sensitivity, 72.7% specificity, and 0.766 AUC. Subsequent analysis to distinguish between CN and LMCI, MCI group (EMCI+LMCI), AD, and patient group (EMCI+LMCI+AD) also showed similar results to those presented in Supp. Table 2.

## Discussion

In this study, we investigated the early features of EMCI using cortical radiality, which reflects mesoscopic structural changes. By leveraging radiality in the gray matter, we could detect the changes in EMCI that were not detected using conventional MRI biomarkers. We found progressively larger regions of decreased radiality as the disease progressed, starting from the medial temporal cortex in EMCI to the whole brain in AD. However, CTh or MD did not show significant differences between CN and EMCI. Furthermore, the radiality results from CN and EMCI non-converter showed similar patterns to those of CN amyloid-negative and -positive (Supp. Figure 1). Based on our results, the microstructural gray matter changes in the bilateral insular cortex are associated with disease progression as seen in the CN and EMCI converter results.

We investigated the relationship between radiality and other imaging measures. The association between radiality and CTh showed a strong positive correlation in various regions of the brain, as shown in Fig. 4. It is clear that a higher CTh indicates a deeper cortical structure, and fiber orientation tends to have a radial orientation. Cortical depth profile analysis showed that thicker cortex was related to larger radiality [28]. In addition, MD showed a strong negative correlation in the temporal, parietal, and frontal cortices. Radiality may be sensitive to CTh but also reflects microstructural features. With AV45 and AV1451, radiality showed an association that widely overlapped with both CTh and MD. Thus, radiality may also reflect changes due to pathologic protein accumulation within the cortex.

Although microstructural changes associated with radiality are unclear, one plausible feature is the disorganization of tangential cortical fibers. It has been reported that tangential cortical fibers develop during neurodevelopment and aging [22]. There are several events that lead to an increase in tangentially oriented fibers, including dendritic elaboration [29], formation of local circuits [30], expansion of thalamo-cortical fibers [31], and disappearance of radial glia [32, 33]. A decrease in radiality may be contrary to those of neurodevelopment. For instance, synaptic loss, neuronal soma changes and neurite disorganization, along with neuronal loss, may lead to a decrease in radiality. These changes may be concurrent with the net loss of macromolecules that affect diffusivity, increasing free water in the extracellular space. However, radiality provides evidence of neuronal density that explains concurrent cortical atrophy. Furthermore, accumulation of amyloid or tau proteins may also participate in the disruption of the microstructure. Given that radiality can identify EMCI, we can further speculate that these microstructural changes occur in the earlier stages of AD, which are not apparent in macroscopic investigation.

To test the sensitivity of radiality, we sought to identify the earliest stage of AD. Interestingly, our CN vs. EMCI radiality analysis did not show a biphasic trajectory, as discussed in a previous work [34]. Thus, we conducted an additional analysis on amyloid-negative CN and amyloid-positive CN (Supp Fig. 1). We observed biphasic behavior for CTh and MD, where biomarkers showed opposite directions of changes. While CTh increased and MD decreased, radiality showed a monotonous decrease in amyloid-positive CN. This distinct behavior of radiality could characterize the changes in EMCI, while CTh and MD could not. Both the CTh increase and MD decrease in the early stage of AD were thought to be caused by an amyloid-induced inflammatory response [13]. However, radiality seems to decrease whenever there are microstructural changes in the tissue. In a preterm study, the occipital cortex showed a decrease in radiality as in early development [19–21]. In the case of multiple sclerosis, decreased radiality was observed in the dorsolateral prefrontal cortex, Heschl’s gyrus, and primary visual cortex, possibly due to cortical alterations [18].

We performed a simple cutoff binary classification analysis to assess the diagnostic accuracy of radiality. The target mask was obtained from the group comparison result of CN versus EMCI, and the individual mean radiality within the mask was used as a classification feature. With varying cutoff values, the model showed 70.5% accuracy with 0.766 AUC to differentiate CN and EMCI, 67.9% with 0.757 AUC for CN and LMCI, 70.5% with 0.766 AUC for CN and MCI (EMCI+LMCI), and 78.6% with 0.867 AUC to CN and AD, as presented in Supp. Table 2. The results were comparable to those of previous studies. A recent study that adopted a logistic regression model with neurite density index, orientation dispersion index, and CTh as features reported 0.72 AUC for CN and MCI and 0.91 AUC for CN and AD [35]. Other studies employing whole MD and gray matter map reported 79.6% accuracy with 0.84 AUC for CN and MCI and 93.5% with 0.94 AUC to CN and AD [36], 76% with 0.83 AUC for NC and AD [37].

There were several limitations to the current study. First, the relatively poor resolution of DTI compared to structural T1 images could lead to inaccurate results. Although surface analysis was employed to mitigate registration or segregation errors, a higher resolution DTI would be needed to observe precise cortical changes. Second, the use of multi-protocol DTI images could influence the observation of progressive changes in MCI. We sought to control for age, gender, years of education, and MRI center between the group while applying harmonization to minimize the variation between subjects [38]. Third, the number of subjects who underwent tau PET imaging was not enough to identify any relationship with tau pathology. In order to focus on progressive changes, not only showing a relationship with amyloid but also with tau is an important aspect [39]. However, several subjects in this study underwent screening only once without follow-up or only MRI data were available.

## Conclusions

In conclusion, we investigated the cortical changes in EMCI using structural MRI, DTI, and PET imaging markers. Only radiality could delineate the changes in EMCI while cortical thickness and MD could not. In addition, radiality changes in the frontal cortex as well as amyloid deposits in the continuum. These results indicate that the multimodal approach, atrophy and microstructure, may illuminate early changes in AD. However, further studies are needed to support the relationship between alterations in cortical structure and diffusion orientation.

## Supplementary information

**Additional file 1: Supplementary Table 1.** Demographics of CN amyloid positivity analysis. **Supplementary Table 2.** Results of cutoff analysis. **Supplementary Fig. 1.** Comparison of amyloid-negative CN and amyloid-positive CN. Group differences in radiality, cortical thickness, and mean diffusivity from CN amyloid-negative and -positive. Radiality showed a decrease in the postcentral cortex, CTh showed an increase in the lingual cortex, and MD showed a decrease in the postcentral cortex. Color bar indicates *p*-value interval of 0.05 to 10^− 5^.

## Data Availability

The MRI and PET data were downloaded from the Alzheimer’s Disease Neuroimaging Initiative (ADNI) database (http://adni.loni.usc.edu/). Application for access to the ADNI data can be submitted by anyone at http://adni.loni.usc.edu/data-samples/access-data/. The process includes completion of an online application form and acceptance of Data Use Agreement.

## Abbreviations

AD: Alzheimer’s disease
ADNI: Alzheimer’s Disease Neuroimaging Initiative
CN: Cognitively normal
CSF: Cerebrospinal fluid
CTh: Cortical thickness
DTI: Diffusion tensor imaging
EMCI: Early mild cognitive impairment
GCDR: Global clinical dementia ratings
LMCI: Late mild cognitive impairment
MADAS-Cog: Modified Alzheimer’s disease Assessment Scale cognitive subscale
MD: Mean diffusivity
MMSE: Mini Mental State Examination
MRI: Magnetic resonance imaging
PET: Positron emission tomography
ROI: Region of interest
SUVR: Standardized uptake value ratio
