# Supplementary material for: Detection of gray matter microstructural changes in Alzheimer’s disease continuum using fiber orientation
Source: BMC Neurol. 2020 Oct 2;20:362. doi: 10.1186/s12883-020-01939-2 (PMC7532608; doi:10.1186/s12883-020-01939-2)
Supplement: Supplementary file 1 — Additional file 1: Supplementary Table 1. Demographics of CN amyloid positivity analysis. Supplementary Table 2. Results of cutoff analysis. Supplementary Fig. 1. Comparison of amyloid-negative CN and amyloid-positive CN. Group differences in radiality, cortical thickness, and mean diffusivity from CN amyloid-negative and -positive. Radiality showed a decrease in the postcentral cortex, CTh showed an increase in the lingual cortex, and MD showed a decrease in the postcentral cortex. Color bar indicates p-value interval of 0.05 to 10− 5. [file 12883_2020_1939_MOESM1_ESM.docx]

**Supplementary information**

**Supplementary Table 1.** Demographics of CN amyloid positivity analysis

|  | **CN Aβ- (n=78)** | **CN Aβ+ (n=28)** |
| --- | --- | --- |
| **Female, n (%)** | 42 (53.8) | 20 (71.4) |
| **Age (SD) (y)** | 72.7±5.9 | 75.1±4.86 |
| **Education (SD) (y)** | 16.7±2.5 | 16.4±2.6 |
| **GCDR (SD)** | 0.0 | 0.0 |
| **MMSE (SD)** | 29.3±1.5 | 29.0±1.0 |
| **MADAS-Cog (SD)** | 9.7±6.8 | 10.6±4.4 |
| **Logical memory I:  Immediate recall (SD)** | 14.2±2.9 | 13.8±3.0 |
| **Logical memory II:  Delayed recall (SD)** | 12.8±3.4 | 13.0±3.1 |
| **Subjects with  Amyloid positive** | 0 (0) | 28 (100) |


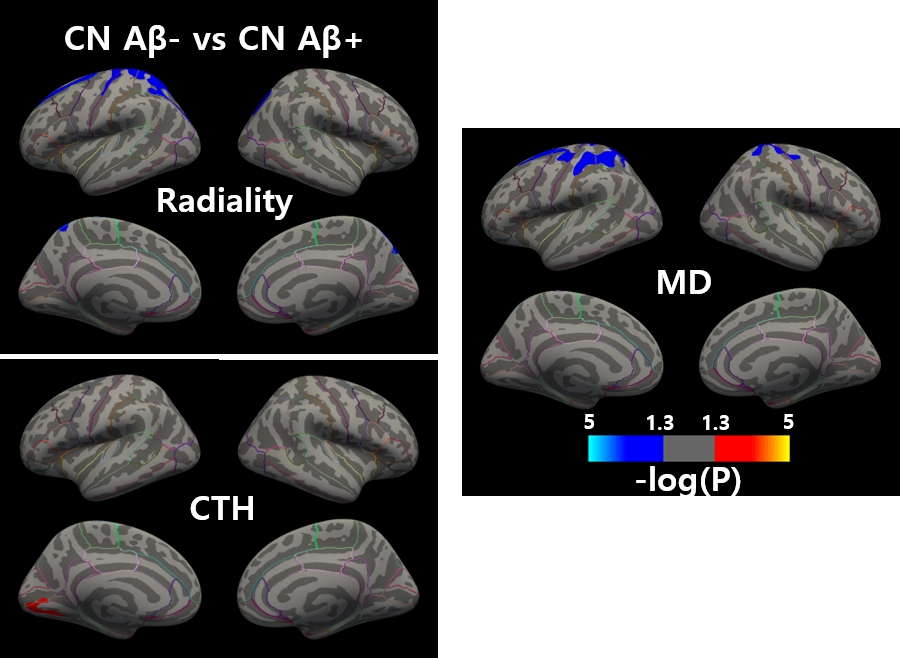


**Supplementary Figure 1.** Comparison of amyloid negative CN and amyloid positive CN

Group difference in radiality, cortical thickness and mean diffusivity from cognitive normal amyloid negative and positive. Radiality showed decrease in left postcentral, CTh showed increase in left lingual, and MD showed decrease in postcentral cortex. Color bar indicates *p*-value interval of 0.05 to 10^-5^

**Supplementary Table 2.** Results of cutoff analysis

|  | **N** | **Accuracy** | **Sensitivity** | **Specificity** | **AUC** |
| --- | --- | --- | --- | --- | --- |
| **CN vs EMCI** | 78 vs 50 | 70.5% | 70.2% | 72.7% | 0.766 |
| **CN vs LMCI** | 78 vs 34 | 67.9% | 70.6% | 66.7% | 0.757 |
| **CN vs (EMCI+LMCI)** | 78 vs 84 | 70.5% | 69.4% | 66.7% | 0.766 |
| **CN vs AD** | 78 vs 39 | 78.6% | 79.5% | 78.1% | 0.867 |
| **CN vs (EMCI+LMCI+AD)** | 78 vs 123 | 72.8% | 72.3% | 73.1% | 0.798 |

**Ethical approval and consent to participate**

The study procedures were approved by the institutional review boards of all participating centers (https://adni.loni.usc.edu/wp-content/uploads/how_to_apply/ ADNI_Acknowledgement_List.pdf), and written informed consent was obtained from all participants or their authorized representatives. Ethics approval was obtained from the institutional review boards of each institution involved: Oregon Health and Science University; University of Southern California; University of California—San Diego; University of Michigan; Mayo Clinic, Rochester; Baylor College of Medicine; Columbia University Medical Center; Washington University, St. Louis; University of Alabama at Birmingham; Mount Sinai School of Medicine; Rush University Medical Center; Wien Center; Johns Hopkins University; New York University; Duke University Medical Center; University of Pennsylvania; University of Kentucky; University of Pittsburgh; University of Rochester Medical Center; University of California, Irvine; University of Texas Southwestern Medical School; Emory University; University of Kansas, Medical Center; University of California, Los Angeles; Mayo Clinic, Jacksonville; Indiana University; Yale University School of Medicine; McGill University, Montreal-Jewish General Hospital; Sunnybrook Health Sciences, Ontario; U.B.C.Clinic for AD & Related Disorders; Cognitive Neurology—St. Joseph’s, Ontario; Cleveland Clinic Lou Ruvo Center for Brain Health; Northwestern University; Premiere Research Inst (Palm Beach Neurology); Georgetown University Medical Center; Brigham and Women’s Hospital; Stanford University; Banner Sun Health Research Institute; Boston University; Howard University; Case Western Reserve University; University of California, Davis—Sacramento; Neurological Care of CNY; Parkwood Hospital; University of Wisconsin; University of California, Irvine—BIC; Banner Alzheimer’s Institute; Dent Neurologic Institute; Ohio State University; Albany Medical College; Hartford Hospital, Olin Neuropsychiatry Research Center; Dartmouth-Hitchcock Medical Center; Wake Forest University Health Sciences; Rhode Island Hospital; Butler Hospital; UC San Francisco; Medical University South Carolina; St. Joseph’s Health Care Nathan Kline Institute; University of Iowa College of Medicine; Cornell University; and University of South Florida: USF Health Byrd Alzheimer’s Institute.
